# Suberoylanilide hydroxamic acid, a histone deacetylase inhibitor, attenuates postoperative cognitive dysfunction in aging mice

**DOI:** 10.3389/fnmol.2015.00052

**Published:** 2015-09-23

**Authors:** Min Jia, Wen-Xue Liu, He-Liang Sun, Yan-Qing Chang, Jiao-Jiao Yang, Mu-Huo Ji, Jian-Jun Yang, Chen-Zhuo Feng

**Affiliations:** ^1^Department of Anesthesiology, Jinling Hospital, School of Medicine, Nanjing UniversityNanjing, China; ^2^Institute of Aging Research, School of Medicine, Hangzhou Normal UniversityHangzhou, China; ^3^Jiangsu Province Key Laboratory of Anesthesiology, Xuzhou Medical CollegeXuzhou, China; ^4^Jiangsu Province Key Laboratory of Anesthesia and Analgesia Application TechnologyXuzhou, China

**Keywords:** postoperative cognitive dysfunction, aging, histone acetylation, neuroapoptosis, synaptic plasticity

## Abstract

Postoperative cognitive dysfunction (POCD) is a recognized clinical entity characterized with cognitive deficits after anesthesia and surgery, especially in aged patients. Previous studies have shown that histone acetylation plays a key role in hippocampal synaptic plasticity and memory formation. However, its role in POCD remains to be determined. Here, we show that suberoylanilide hydroxamic acid (SAHA), a histone deacetylase inhibitor, attenuates POCD in aging Mice. After exposed to the laparotomy, a surgical procedure involving an incision into abdominal walls to examine the abdominal organs, 16- but not 3-month old male C57BL/6 mice developed obvious cognitive impairments in the test of long-term contextual fear conditioning. Intracerebroventricular (i.c.v.) injection of SAHA at the dose of (20 μg/2 μl) 3 h before and daily after the laparotomy restored the laparotomy-induced reduction of hippocampal acetyl-H3 and acetyl-H4 levels and significantly attenuated the hippocampus-dependent long-term memory (LTM) impairments in 16-month old mice. SAHA also reduced the expression of cleaved caspase-3, inducible nitric oxide synthase (iNOS) and N-methyl-D-aspartate (NMDA) receptor-calcium/calmodulin dependent kinase II (CaMKII) pathway, and increased the expression of brain-derived neurotrophic factor (BDNF), synapsin 1, and postsynaptic density 95 (PSD95). Taken together, our data suggest that the decrease of histone acetylation contributes to POCD and may serve as a target to improve the neurological outcome of POCD.

## Introduction

Postoperative cognitive dysfunction (POCD) is a cognitive progressive deterioration in memory and concentration following exposure to anesthesia and surgery (Amar et al., 1998; Terrando et al., 2011; Hovens et al., 2012). These cognitive deficits result in prolonged hospitalization and decreased quality of life (Moller et al., 1998). Tissue damage induced neuro-inflammation and altered reactivity of the immune system after operation are considered to play a major role in the development of POCD, which elicits neuron damages, affects synaptic function, and thereby induces cognitive impairments (Wan et al., 2007; Fidalgo et al., 2011). However, the molecular mechanisms underlying POCD remain largely to be determined.

Epigenetic dysregulation on the expression of key genes is widely involved in the etiology of brain disorders, including Alzheimer’s disease (AD), Huntington’s disease, Parkinson’s disease, and Rubinstein-Taybi syndrome (Petrij et al., 1995; Kazantsev and Thompson, 2008; Chuang et al., 2009; Francis et al., 2009; Peleg et al., 2010; Gräff and Tsai, 2013). Histone acetylation is one of the most common forms of epigenetic modification, which is controlled by the balance between histone acetyltransferases (HATs) and histone deacetylases (HDACs; Fischer et al., 2010; Haggarty and Tsai, 2011; McQuown et al., 2011). In general, histone acetylation facilitates gene transcription, whereas histone deacetylation results in gene silencing (Fischer et al., 2010; Haggarty and Tsai, 2011; McQuown et al., 2011). A substantial body of evidence suggests that the dysregulation of histone acetylation contributes to the pathogenesis of neurodegenerative diseases, and targeted restoration of histone acetylation by HDAC inhibitors shows neuroprotective effects on neurodegenerative diseases (Petrij et al., 1995; Dash et al., 2010; Kilgore et al., 2010; Haettig et al., 2011; Ji et al., 2014).

The similar clinical symptoms has been revealed between POCD and neurodegenerative disorders (Wang et al., 2013; Luo et al., 2014; Xu et al., 2014). However, comparing with the studies of neurodegenerative diseases, the potential function of histone acetylation in POCD remains primarily unknown. Therefore, based on the pre-clinical animal mode of the laparotomy-induced cognitive deficits (Rosczyk et al., 2008; Barrientos et al., 2012; Hovens et al., 2014), which surgical procedure involving an incision into the abdominal wall to examine the abdominal organs, we investigated the role of histone acetylation and potential therapeutic effect of an HDAC inhibitor, suberoylanilide hydroxamic acid (SAHA), on POCD.

## Materials and Methods

### Animals

All animal experiments were carried out in accordance with the National Institutes of Health Guide for the Care and Use of Laboratory Animals, USA. The study protocol was approved by the Institutional Animal Care and Use Ethics Committee, Jinling Hospital, Nanjing University, Nanjing, China. The mice were purchased from The Animal Center of Jinling Hospital, Nanjing, China and efforts were made to minimize the number of animals used and their suffering. The mice were housed under specific pathogen-free conditions in a temperature-controlled room of 23 ± 1°C on a 12-h light-dark cycle, with *ad libitum* access to food and water. Mice were allowed 7 days to acclimate to the laboratory conditions before experiments.

### Study Groups of Animals

**Figure 1:**
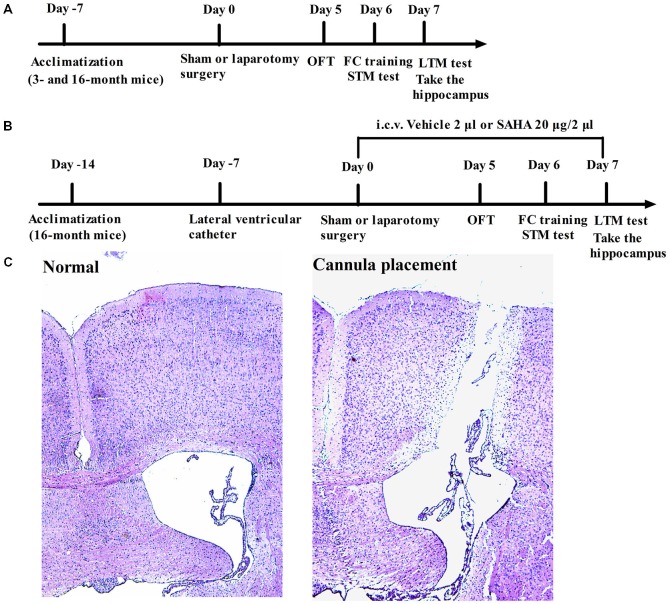
**Diagrammatic presentation of the experimental protocol (A,B) and the position of intracerebroventricular (i.c.v.) cannulation confirmed by the hematoxylin-eosin (HE) staining in the 16-month old mice (C)**.

In the first set of experiments, the 3- and 16-month old male C57BL/6 mice were used. Thirty-two 3-month old mice weighing 25–32 g and thirty-two 16-month old mice weighing 33–40 g were randomly assigned to receiving laparotomy or sham surgery (*n* = 16 for each group). The experimental protocol was presented in Figure 1A.

In the second set of experiments with SAHA (a histone deacetylase inhibitor) treatment, sixty-four 16-month old male mice weighing 33–40 g were randomly assigned to the following four groups: Sham + Vehicle group, mice received vehicle treatment and sham surgery; Sham + SAHA group, mice received SAHA treatment and sham surgery; Laparotomy + Vehicle group, mice received vehicle treatment and laparotomy; and Laparotomy + SAHA group, mice received SAHA treatment and laparotomy (*n* = 16 for each group). The experimental protocol was presented in Figure 1B.

### Surgery

The Laparotomy or sham surgery was performed as previously described (Rosczyk et al., 2008; Barrientos et al., 2012; Hovens et al., 2014). Anesthesia was induced with 1.5% isoflurane in 100% oxygen in mice and was still maintained with 1.5% isoflurane by mice anesthesia mask during the surgery procedure. After the abdominal region of mice was shaved and cleaned with iodophor disinfectant, a 1.5 cm vertical incision, approximately 0.5 cm below the lower right rib, was created. The viscera and musculature were vigorously manipulated by inserting a sterile probe into the body cavity and stretching the musculature. Intestine was then exteriorized and manipulated between the surgeon’s thumb and forefinger. The intestines were then placed back into the peritoneal cavity. The surgeries were lasted for approximately 15 min. After that, the peritoneal lining, muscle wall and the skin were closed with three dissolvable sutures and four silk thread sutures, respectively. The exterior wounds were dressed with polysporin to prevent potential infection. To eliminate the effect of hypoxia and acidosis to the experiment, both hypoxia and acidosis were analyzed by using arterial blood gas as we described before Li et al. (2014). Isoflurne anesthesia was stopped immediately for all groups of mice once the suture was in place. After recovery from anesthesia, the mice were placed back into their home cages with *ad libitum* access to food and water. For the sham surgery, mice were anesthetized, shaved, cleaned and the incision was sutured under isoflurane anesthesia for the same duration as those that the laparotomy surgical mice spent. Without manipulation of the viscera or musculature, the incision was closed and treated as described above.

### Cannula Placement and SAHA Treatment

Cannula placement was performed 7 days before the surgery (Figure 1C). In the second set of experiments, each mouse was stereotaxically implanted with 24-gauge, stainless steel guide cannula (RWD Life Science Co., Ltd, Shenzhen, China) under anesthesia with intraperitoneal injection of 50 mg/kg 2% pentobarbital sodium. The position of guide cannula was in the left lateral ventricle, which is 0.45 mm posterior to bregma, 1.08 mm lateral to bregma, and 2.50 mm deep to dura. Once the guide cannula was placed, it was fixed to skull with glass ionomer cement (Dental Materials Factory of Shanghai Medical instrument Co., Ltd, Shanghai, China). The cannula placements were verified by postmortem dissections of brain tissue, followed by hippocampus collections.

The dose and time point for SAHA treatment (S1047, Selleckchem, TX, USA) were designed according to the previous study, in which it was showed that the hippocampal histone acetylation levels peaked 3-h after the intracerebroventricular (i.c.v.) injection of SAHA in mice (Alarcón et al., 2004). SAHA was dissolved in 40% of dimethylsulfoxide (DMSO) diluted with saline. In the group of mice treated with SAHA, the dose of 20 μg in 2 μl was used through i.c.v. injection once daily for 7 days. The first dose was given at the time of 3-h before the laparotomy or sham surgery. For the vehicle controls, the same amount of 40% DMSO was used.

### Hematoxylin-Eosin (HE) Staining

In our preliminary experiment, to ensure the fixed position of left lateral ventricle, 7 days after cannula placement, mice were anesthetized deeply with intraperitoneal injection of 50 mg/kg 2% pentobarbital sodium, and then perfused transcardially with 0.1 M phosphate-buffered saline (PBS, pH 7.4), followed by 4% phosphate-buffered paraformaldehyde (PFA) for tissue fixation. The brain was removed, post-fixed in the same PFA solution for 12 h and sequentially immersed in 30% sucrose-phosphate-buffer solution for 24 h at 4°C. Coronal 5-μm-thick cryostat sections were cut for the routine hematoxylin-eosin (HE) staining.

Each section was stained in Harris’s hematoxylin solution for 8 min, differentiated in 1% acid alcohol for 30 s. After rinsing in 95% alcohol, the slides were counterstained in eosin-phloxine B solution for 45 s. After being dehydrated in a graded series of ethanol and cleared in xylene solutions, the sections were mounted for observation under a light microscope (Olympus BX53F, Tokyo, Japan).

### Open Field Test

All behavioral procedures were performed during the light phase of the cycle between 10:00 A.M. and 4:00 P.M. in a sound-isolated room. Five days after the laparotomy, the mice were subjected to the open field test. The open field apparatus was positioned in a dimly lit room and consisted of a white Plexiglas chamber (40 cm × 40 cm with walls 40 cm high). Each mouse was placed at the center of the arena and left to explore the whole field for 5 min of recording by using the video tracking system (XR-XZ301, Shanghai Softmaze Information Technology Co. Ltd, Shanghai, China). The total distance traveled and the time spent in the center was measured as the parameter of anxiolytic behavior. Between each test, the surface of the arena was thoroughly cleaned with 75% alcohol to avoid the presence of olfactory cues. Tests were recorded by a person who was blinded to the grouping of mice.

### Fear Conditioning Test

On the sixth day after the laparotomy, mice were subjected to fear conditioning test by using the fear conditioning paradigm (XR-XC404, Shanghai Softmaze Information Technology Co. Ltd, Shanghai, China). A mouse was placed in a conditioning training chamber (30 cm × 30 cm with walls 45 cm high) enclosed by a soundproof box with a camera fixed on top. After a 3 min baseline exploratory period in the chamber, mice received one tone (30 s, 70 dB, 3 kHz)-foot-shock (2 s, 0.75 mA) pairing. The foot-shock was carried out at the last 2 s of tone stimulation. Afterward, the mice were left in the conditioning box for additional 30 s before being returned to their home cage. Two hours after the training session, one batch of mice was place again in the training chamber and subjected to the short-term memory (STM) test. During a period of 5 min in the absence of tone and foot shock to test contextual fear conditioning to evaluate hippocampus-dependent memory, the freezing behavior of each mouse was scored every 5 s. Two hours after the contextual fear conditioning test, the mice were placed to a novel chamber for the cued (tone) fear conditioning test to evaluate amygdala-dependent memory. After a 3 min exploratory period in the new chamber, a training tone (30 s, 70 dB, 3 kHz) was applied for another 3 min and freezing behavior was scored during this tone period. The long-term memory (LTM) was performed at the time 24-h after training session and another batch of mice were used. Between each test, the chamber was thoroughly cleaned with 75% alcohol to avoid the presence of olfactory cues. The fear conditioning was administered and evaluated by a person blinded to the group assignment of mice.

### Preparation of Protein Extracts

Two hours after the LTM test, mice were sacrificed and the hippocampus was harvested. The samples for measuring histone acetylation were prepared as described before Kilgore et al. (2010). Briefly, each sample was homogenized in the buffer containing 50 mM Tris-HCl, pH 7.5, 25 mM KCl, 250 mM sucrose, 2 mM sodium butyrate, 1 mM sodium orthovanadate, 0.5 mM PMSF and 1× protease inhibitor cocktail (sigma, MO, USA). After centrifuge at 7700 × g for 1 min at 4°C to pellet nuclei, 0.4 N H_2_SO_4_ was added to the pellet used for separating the histones. Then trichloroacetic acid with 10 mM sodium deoxycholate was added to supernatant to precipitate histone and incubate on ice for 30 min. After centrifuge at 14000 × g for 30 min at 4°C, the pellet of histone was washed once by acidified acetone and then resuspended in 10 mM Tris-HCl, pH 8.0.

For measuring the proteins of inducible nitric oxide synthase (iNOS), brain-derived neurotrophic factor (BNDF), synapsin 1, PSD-95, NR2A, NR2B, calcium/calmodulin dependent kinase II (CaMKIIα), and CaMKIIβ, Radio-Immunoprecipitation Assay (RIPA) buffer containing 1 × protease inhibitor cocktail was used. Homogenates were centrifuged at 13000 × g at 4°C for 10 min and the supernatants were collected for western blot.

### Western Blot

Approximately 1 μg of histone protein or 50 μg of total protein per lane was separately by polyacrylamide gels and then transferred to a polyvinylidene difluoride membrane. After being incubated in blocking buffer of 5% non-fat milk in Tris-Buffered Saline Tween (TBST), membranes were incubated overnight in each primary antibody at 4°C. The primary antibodies used were anti-histone H3 (1:900; Cell Signaling, MA, USA), anti-histone H4 (1:900; Cell Signaling, MA, USA), anti-acetyl histone H3 (1:800; Merck Millipore, Darmstadt, Germany), anti-acetyl histone H4 (Lys5/8/12/16; 1:800; Merck Millipore, Darmstadt, Germany), anti-histone H3 (acetyl K9; 1:800; Abcam, MA, UK), anti-histone H3 (acetyl K14; 1:800; Merck Millipore, Darmstadt, Germany), anti-histone H4 (acetyl K5; 1:800; Abcam, MA, UK), anti-histone H4 (acetyl K12; 1:900; Abcam, MA, UK), anti-Cleaved Caspase-3 (1:900; Cell Signaling, MA, USA), anti-iNOS (1:2000; ANBO, CA, USA), anti-BDNF (1:1500; Santa Cru, CA, USA), anti-Synapsin 1 (1:2500; Merck Millipore, Darmstadt, Germany), anti-postsynaptic density 95 (PSD95) (1:1500; Abcam, MA, UK), anti-NMDAR2A (1:1000; Abcam, MA, UK), anti-NMDAR2B (1:1000; Abcam, MA, UK), anti-CaMKIIα (1:1000; Abcam, MA, UK), anti-CaMKIIβ (1:1000; Abcam, MA, UK). Membranes were washed with TBST and incubated with appropriate secondary antibodies (goat anti-rabbit or goat anti-mouse; Santa Cru, CA, USA). Protein bands were visualized by using enhanced chemiluminescence method and quantitatively analyzed with Image J Quant Software (NIH, Bethesda, MD, USA). The densities of histone acetylation bands were normalized to those of histone from the same sample. The results from various experimental conditions were normalized to the data of mice in the Sham + Vehicle group.

#### Real-Time PCR

Real-time polymerase chain reaction (Real-time PCR) was performed as described previously (Feng et al., 2011). Total RNA was extracted from hippocampus of mouse using RNeasy micro kit (Qiagen, Valencia, CA, USA). Primers for real-time PCR were designed based on the reported sequence of mouse gene iNOS, BDNF, synapsin 1, PSD95, NR2A, NR2B, CaMKIIα, and CaMKIIβ and designed by OligoPerfect Designer. The primers in conserved coding region were preferred, if the gene has various transcripts. The sequences of the primers were detailed in Table 1. Quantitative PCRs were carried out in triplicate using each cDNA sample that was equivalent to 50 ng of stating total RNA. SYBR Green Quantitative PCR protocol was performed by using iQ SYBR Green Supermix (Bio-rad, CA, USA) in the Bio-Rad CFX96 real-time detection system (Bio-rad, CA, USA). To account for the possible differences in staring cDNA, quantitative PCR of the housekeeping genes glyceraldehyde-3-phosphate dehydrogenase (GAPDH) was also carried out for each sample. After PCR reaction, samples were subjected to a temperature ramp (from 70–95°C, 2°C/s) with continuous fluorescence monitoring for melting curve analysis. For each PCR product, a single narrow peak was obtained by melting curve analysis at the specific temperature. The relative amount of mRNA in each sample was determined using the comparative threshold cycle method and then normalized those of housekeeping gene GAPDH.

**Table 1:** **The sequence of primers for real-time PCR analysis**.

Genes	Forward primers (5′-3′)	Reverse primers (5′-3′)	Accession number
iNOS	GGATTGTCCTACACCACACCAA	ATCTCTGCCTATCCGTCTCGTC	NM_010927
BDNF	AGCTGAGCGTGTGTGACAGT	ACCCATGGGATTACACTTGG	NM_007540
Synapsin1	GCTGGAATCCCCAGTGTAAA	AGTTCCACGATGAGCTGCTT	NM_013680
PSD95	CCCCAACATGGACTGTCTCT	ACTCCATCTCCCCCTCTGTT	NM_007864
NR2A	CTCTGATAATCCTTTCCTCCAC	GACCGAAGATAGCTGTCATTTACT	NM_008170
NR2B	TCCATCAGCAGAGGTATCTACAG	CCGTTGACTCCAGACAGGTT	NM_008171
CaMKIIα	GCCTCAGTCCTCCTGTGAAG	ACTCCTCTTCCCACCCACTT	NM_009792
CaMKIIβ	ATCGCCACCGCCATGGCCAC	GGTGATCTCTGGCCGACAGCT	NM_001174053
GAPDH	ACCCAGAAGACTGTGGATGG	CACATTGGGGGTAGGAACAC	NM_001289726

#### Statistical Analysis

Data are presented as the mean ± S.E.M. and analyzed by the Statistical Product for Social Sciences (SPSS; version 17.0, IL, USA). The difference among groups was determined by two-way analysis of variance followed by Bonferroni’s *post hoc* test. Age and surgery type, or surgery type and drug treatment, were considered as two independent factors. The *P* values of age, surgery type, drug and interaction of factors were presented by *P*_age_, *P*_surg_, *P*_drug_ and *P*_int_ respectively. A *P* value < 0.05 was regarded as statistical significance.

## Results

### Laparotomy Induced the Hippocampus-Dependent Long-Term Cognitive Impairments and Down-Regulation of Hippocampal Acetyl-H3 and Acetyl -H4 Levels in the 16- but not the 3-Month Old Mice

**Figure 2:**
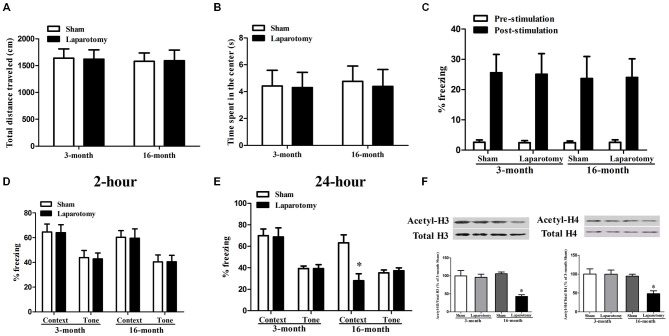
**Impact of the cognition and histone acetylation in the 3- and 16-month old mice after surgery. (A,B)** Performance of total distance traveled and time spent in the center during the open field test. Data are presented as the mean ± S.E.M. (*n* = 16). **(C)** Performance of freezing time during the fear conditioning training session. Data are presented as the mean ± S.E.M. (*n* = 16). **(D,E)** Performance during fear conditioning tests 2- or 24-h after laparotomy. Data are presented as the mean ± S.E.M. (*n* = 8). **(F)** The acetylation level of histone H3 and H4 in the 3- or 16-month-old mice after laparotomy. Results are mean ± S.E.M. (*n* = 3). *p < 0.05 compared with the 16-month old mice subjected to sham surgery.

To investigate the difference of POCD between adult and aging mice, laparotomy or sham surgery was performed on the 3- and 16-month old mice. Neither the 3- nor the 16-month old mice had significant difference in the total distance traveled (*P*_age_ = 0.804, *F*_age(1,28)_ = 0.0626; *P*_surg_ = 0.982, *F*_surg(1,28)_ = 0.00053; *P*_int_ = 0.913, *F*_int(1,28)_ = 0.00761) or time spent in the center (*P*_age_ = 0.855, *F*_age(1,28)_ = 0.0338; *P*_surg_ = 0.839, *F*_surg(1,28)_ = 0.0421; *P*_int_ = 0.913, *F*_int(1,28)_ = 0.0122) after the laparotomy or sham surgery (Figures 2A,B). Then we used fear conditioning test to determine the associative memory. The laparotomy did not induce the acquirement of associative memory during the training session of pre-stimulation (*P*_age_ = 0.957, *F*_age(1,28)_ = 0.00295; *P*_surg_ = 0.985, *F*_surg(1,28)_ = 0.000353; *P*_int_ = 0.843, *F*_int(1,28)_ = 0.0398) or post-stimulation (*P*_age_ = 0.825, *F*_age(1,28)_ = 0.0498; *P*_surg_ = 0.990, *F*_surg(1,28)_ = 0.000176; *P*_int_ = 0.948, *F*_int(1,28)_ = 0.00432; Figure 2C). In the STM test, no significant difference was found in the context (*P*_age_ = 0.506, *F*_age(1,28)_ = 0.454; *P*_surg_ = 0.920, *F*_surg(1,28)_ = 0.0103; *P*_int_ = 0.994, *F*_int(1,28)_ = 0.0000547) or tone test (*P*_age_ = 0.593, *F*_age(1,28)_ = 0.293; *P*_surg_ = 0.925, *F*_surg(1,28)_ = 0.00893; *P*_int_ = 0.916, *F*_int(1,28)_ = 0.0113) among the four groups (Figure 2D). However, in the LTM test, the laparotomy led to a shorter freezing time in the context (*P*_age_ = 0.003, *F*_age(1,28)_ = 10.750; *P*_surg_ = 0.017, *F*_surg(1,28)_ = 6.396; *P*_int_ = 0.025, *F*_int(1,28)_ = 5.583) but not in the tone test (*P*_age_ = 0.301, *F*_age(1,28)_ = 1.113; *P*_surg_ = 0.737, *F*_surg(1,28)_ = 0.115; *P*_int_ = 0.736, *F*_int(1,28)_ = 0.116) compared with the sham surgery in 16-month, but not 3-month old mice (Figure 2E).

We next examined the levels of acetyl-H3 and acetyl-H4 that are deeply involved in neurodegeneration diseases (Guan et al., 2009; Ricobaraza et al., 2009; Castellano et al., 2012). We found that the cognitive impairments in the 16-month old mice was accompanied with a decreased level of hippocampal acetyl-H3 (*P*_age_ = 0.034, *F*_age(1,8)_ = 6.485; *P*_surg_ = 0.007, *F*_surg(1,8)_ = 12.971; *P*_int_ = 0.013, *F*_int(1,8)_ = 10.043) and acetyl-H4 (*P*_age_ = 0.020, *F*_age(1,8)_ = 8.301; *P*_surg_ = 0.047, *F*_surg(1,8)_ = 5.499; *P*_int_ = 0.049, *F*_int(1,8)_ = 5.359), whereas the 3-month old mice whose memory were not damaged did not show such a reduction (Figure 2F).

### SAHA Ameliorated the Hippocampus-Dependent Long-Term Cognitive Impairments in the 16-Month Old Mice Exposed to the Laparotomy

**Figure 3:**
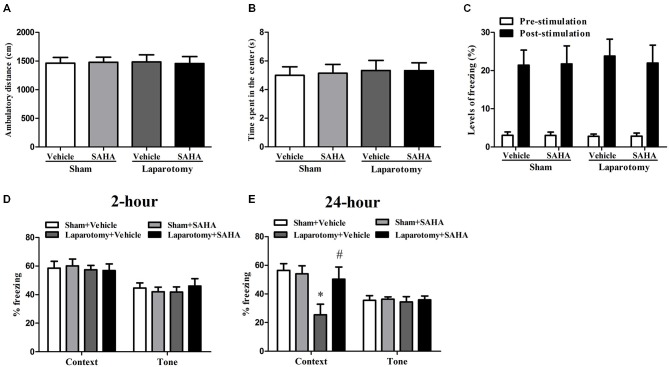
**Impact of suberoylanilide hydroxamic acid (SAHA) treatment on the cognitive performance in the 16-month old mice after surgery. (A,B)** Performance of total distance traveled and time spent in the center during the open field test. Data are presented as the mean ± S.E.M. (*n* = 16). **(C)** Performance of freezing time during the fear conditioning training session. Data are presented as the mean ± S.E.M. (*n* = 16). **(D,E)** Performance during the fear conditioning tests 2- or 24-h after laparotomy. Data are presented as the mean ± S.E.M. (*n* = 8). *p < 0.05 compared with the Sham + Vehicle group; ^#^*p* < 0.05 compared with the Laparotomy + Vehicle group.

The poor hippocampus-dependent LTM with histone acetylation down-regulation in the 16-month old mice exposed to the surgery guided us to further investigate the effect of SAHA on these mice. The results didn’t showed any significant differences in the total distance traveled (*P*_surg_ = 0.984, *F*_surg(1,60)_ = 0.000388; *P*_drug_ = 0.955, *F*_drug(1,60)_ = 0.00318; *P*_int_ = 0.847, *F*_int(1,60)_ = 0.0375), time spent in the center (*P*_surg_ = 0.676, *F*_surg(1,60)_ = 0.177; *P*_drug_ = 0.904, *F*_drug(1,60)_ = 0.0148; *P*_int_ = 0.899, *F*_int(1,60)_ = 0.0163), or ability of memory acquirement when calculating the freezing time in the pre-stimulation (*P*_surg_ = 0.984, *F*_surg(1,60)_ = 0.000388; *P*_drug_ = 0.955, *F*_drug(1,60)_ = 0.00318; *P*_int_ = 0.847, *F*_int(1,60)_ = 0.0375) and post-stimulation (*P*_surg_ = 0.774, *F*_surg(1,60)_ = 0.0833; *P*_drug_ = 0.866, *F*_drug(1,60)_ = 0.0287; *P*_int_ = 0.812, *F*_int(1,60)_ = 0.0573) among the four groups (Figures 3A–C). In the STM test, no significant difference was found in the context (*P*_surg_ = 0.620, *F*_surg(1,28)_ = 0.251; *P*_drug_ = 0.905, *F*_drug(1,28)_ = 0.0146; *P*_int_ = 0.816, *F*_int(1,28)_ = 0.0554) or tone test (*P*_surg_ = 0.872, *F*_surg(1,28)_ = 0.0265; *P*_drug_ = 0.837, *F*_drug(1,28)_ = 0.0429; *P*_int_ = 0.403, *F*_int(1,28)_ = 0.721) among the four groups (Figure 3D). In the LTM test, the percent of freezing time in the context test decreased in the Laparotomy + Vehicle group compared with the Sham + Vehicle group, whereas SAHA diminished the decrease in the Laparotomy + SAHA group compared with the Laparotomy + Vehicle group (*P*_surg_ = 0.014, *F*_surg(1,28)_ = 6.802; *P*_drug_ = 0.049, *F*_drug(1,28)_ = 4.254; *P*_int_ = 0.021, *F*_int(1,28)_ = 5.991; Figure 3E). No significant difference was observed in the tone test of LTM test among the four groups (*P*_surg_ = 0.803, *F*_surg(1,28)_ = 0.0632; *P*_drug_ = 0.701, *F*_drug(1,28)_ = 0.150; *P*_int_ = 0.898, *F*_int(1,28)_ = 0.0167; Figure 3E).

### SAHA Restored the Down-Regulation of Hippocampal Histone Acetylation in the 16-Month Old Mice Exposed to the Laparotomy

**Figure 4:**
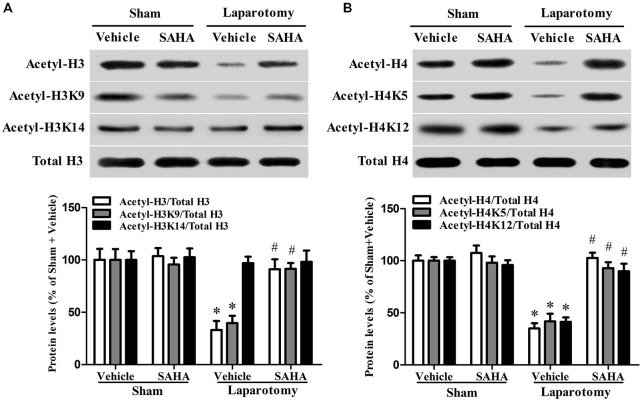
**Impact of SAHA treatment on the level of histone acetylation in the 16-month old mice after surgery. (A)** The representative blot of histone H3 acetylation is shown at the top of the panel and the corresponding quantitative result is shown at the bottom. Data are presented as the mean ± S.E.M. (*n* = 3). **(B)** The representative blot of histone H4 acetylation is shown at the top of the panel and the corresponding quantitative result is shown at the bottom. Data are presented as the mean ± S.E.M. (*n* = 3). *p < 0.05 compared with the Sham + Vehicle group; ^#^*p* < 0.05 compared with the Laparotomy + Vehicle group.

The hippocampal levels of acetyl-H3 (*P*_surg_ = 0.003, *F*_surg(1,8)_ = 18.703; *P*_drug_ = 0.010, *F*_drug(1,8)_ = 11.213; *P*_int_ = 0.018, *F*_int(1,8)_ = 8.799) and acetyl-H4 (*P*_surg_ < 0.001, *F*_surg(1,8)_ = 38.201; *P*_drug_ < 0.001, *F*_drug(1,8)_ = 44.448; *P*_int_ < 0.001, *F*_int(1,8)_ = 28.585) decreased in the Laparotomy + Vehicle group than those in the Sham + Vehicle group, whereas SAHA abolished the decrease in the Laparotomy + SAHA group compared with the Laparotomy + Vehicle group (Figures 4A,B). Four associated acetylation sites including acetyl-H3K9, acetyl-H3K14, acetyl-H4K5, and acetyl-H4K12 were analyzed. The hippocampal acetylation levels of H3K9 (*P*_surg_ = 0.005, *F*_surg(1,8)_ = 14.682; *P*_drug_ = 0.007, *F*_drug(1,8)_ = 12.972; *P*_int_ = 0.006, *F*_int(1,8)_ = 13.898), H4K5 (*P*_surg_ < 0.001, *F*_surg(1,8)_ = 29.653; *P*_drug_ = 0.003, *F*_drug(1,8)_ = 17.851; *P*_int_ = 0.002, *F*_int(1,8)_ = 20.698), and H4K12 (*P*_surg_ < 0.001, *F*_surg(1,8)_ = 40.116; *P*_drug_ = 0.002, *F*_drug(1,8)_ = 19.008; *P*_int_ < 0.001, *F*_int(1,8)_ = 26.543) decreased in the Laparotomy + Vehicle group compared with the Sham + Vehicle group, whereas SAHA blocked the decreases in the Laparotomy + SAHA group compared with the Laparotomy + Vehicle group (Figures 4A,B). No significant difference was observed in the level of acetyl-H3K14 among the four groups (*P*_surg_ = 0.169, *F*_surg(1,8)_ = 2.288; *P*_drug_ = 0.588, *F*_drug(1,8)_ = 0.319; *P*_int_ = 0.829, *F*_int(1,8)_ = 0.0501; Figures 4A,B).

### SAHA Prevented the Dysregulation of Hippocampal Neuroapoptosis- and Synaptic Plasticity-Related Proteins in the 16-Month Old Mice Exposed to the Laparotomy

**Figure 5:**
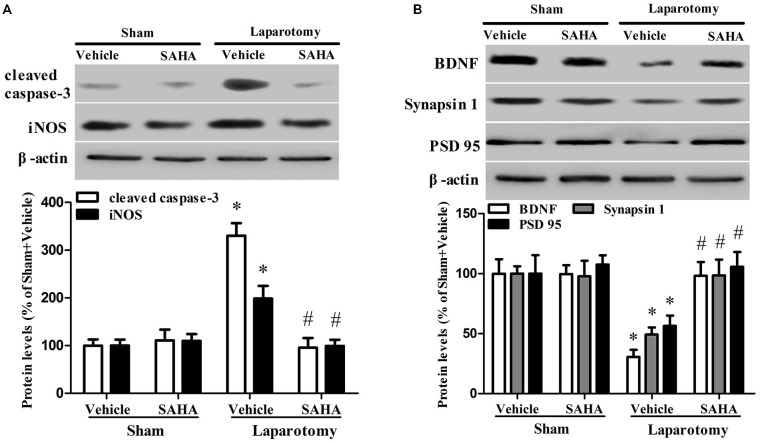
**Impact of SAHA treatment on protein expressions of cleaved caspase-3, iNOS, BDNF, synapsin 1 and PSD95 in the 16-month old mice after surgery. (A)** The protein levels of cleaved caspase-3 and iNOS were determined by western blot. Representative image is at the top and quantitative result is at the bottom. Data are presented as the mean ± S.E.M. (*n* = 3). **(B)** The result of western blot for BDNF, synapsin 1, and PSD95. Representative image is shown at the top and quantitation is at the bottom. Data are presented as the mean ± S.E.M. (*n* = 3). *p < 0.05 compared with the Sham + Vehicle group; ^#^*p* < 0.05 compared with the Laparotomy + Vehicle group.

The hippocampal protein levels of cleaved caspase-3 (*P*_surg_ < 0.001, *F*_surg(1,8)_ = 49.285; *P*_drug_ < 0.001, *F*_drug(1,8)_ = 37.549; *P*_int_ < 0.001, *F*_int(1,8)_ = 38.197) and iNOS (*P*_surg_ = 0.038, *F*_surg(1,8)_ = 6.139; *P*_drug_ = 0.036, *F*_drug(1,8)_ = 6.356; *P*_int_ = 0.015, *F*_int(1,8)_ = 9.508) increased in the Laparotomy + Vehicle group compared with the Sham + Vehicle group, whereas SAHA eliminated the increases in the Laparotomy + SAHA group compared with the Laparotomy + Vehicle group (Figure 5A). The hippocampal protein levels of BDNF (*P*_surg_ = 0.010, *F*_surg(1,8)_ = 11.409; *P*_drug_ = 0.007, *F*_drug(1,8)_ = 13.191; *P*_int_ = 0.006, *F*_int(1,8)_ = 13.434), synapsin 1 (*P*_surg_ = 0.039, *F*_surg(1,8)_ = 6.070; *P*_drug_ = 0.049, *F*_drug(1,8)_ = 5.387; *P*_int_ = 0.035, *F*_int(1,8)_ = 6.418), and PSD95 (*P*_surg_ = 0.024, *F*_surg(1,8)_ = 7.708; *P*_drug_ = 0.060, *F*_drug(1,8)_ = 4.786; *P*_int_ = 0.069, *F*_int(1,8)_ = 4.401) decreased in the Laparotomy + Vehicle group compared with the Sham + Vehicle group, whereas SAHA reversed the decreases in the Laparotomy + SAHA group compared with the Laparotomy + Vehicle group (Figure 5B).

**Figure 6:**
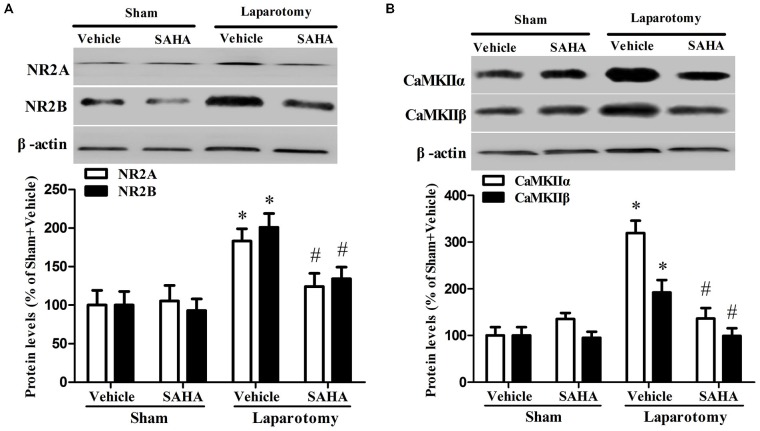
**Impact of SAHA treatment on protein expressions of NR2A, NR2B, CaMKIIα, and CaMKIIβ in the 16-month old mice after surgery. (A)** The protein levels of NR2A, NR2B were determined by western blot. Representative image is shown at the top and quantitative result at the bottom. Data are presented as the mean ± S.E.M. (*n* = 3). **(B)** The protein levels of CaMKIIα and CaMKIIβ. Representative image is shown at the top and quantitative result at the bottom. Data are presented as the mean ± S.E.M. (*n* = 3). *p < 0.05 compared with the Sham + Vehicle group; ^#^*p* < 0.05 compared with the Laparotomy + Vehicle group.

**Figure 7:**
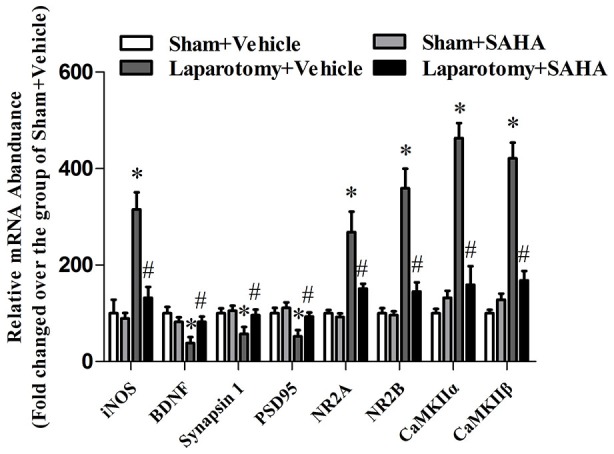
**Impact of SAHA treatment on mRNA abundances of iNOS, BDNF, synapsin 1, PSD95, NR2A, NR2B, CaMKIIα, and CaMKIIβ in the 16-month old mice after surgery.** The real-time PCR results were normalized by those of genes glyceraldehyde-3-phosphate dehydrogenase (GAPDH). The results from each group were then normalized by those from the group of vehicle-treated mice subjected to sham surgery. Data are presented as the mean ± S.E.M. (*n* = 3). *p < 0.05 compared with the Sham + Vehicle group; ^#^*p* < 0.05 compared with the Laparotomy + Vehicle group.

The hippocampal NR2A (*P*_surg_ = 0.007, *F*_surg(1,8)_ = 12.823; *P*_drug_ = 0.048, *F*_drug(1,8)_ = 5.428; *P*_int_ = 0.029, *F*_int(1,8)_ = 7.055), NR2B (*P*_surg_ < 0.001, *F*_surg(1,8)_ = 26.202; *P*_drug_ = 0.025, *F*_drug(1,8)_ = 7.533; *P*_int_ = 0.037, *F*_int(1,8)_ = 6.217), CaMKIIα (*P*_surg_ < 0.001, *F*_surg(1,8)_ = 28.352; *P*_drug_ = 0.007, *F*_drug(1,8)_ = 12.744; *P*_int_ < 0.001, *F*_int(1,8)_ = 27.711), and CaMKIIβ (*P*_surg_ = 0.037, *F*_surg(1,8)_ = 6.203; *P*_drug_ = 0.020, *F*_drug(1,8)_ = 8.463; *P*_int_ = 0.029, *F*_int(1,8)_ = 7.001) were up-regulated in the Laparotomy +Vehicle group compared with the Sham + Vehicle group, whereas SAHA inhibited the up-regulation in the Laparotomy + SAHA group compared with the Laparotomy + Vehicle group (Figure 6). The gene expression level changes at the protein level measured by western blot were consistent to those at mRNA level measured by RT-real time PCR (Figure 7). The statistical results were as follows: iNOS (*P*_surg_ < 0.001, *F*_surg(1,8)_ = 89.695; *P*_drug_ < 0.001, *F*_drug(1,8)_ = 50.714; *P*_int_ < 0.001, *F*_int(1,8)_ = 39.864), BDBF (*P*_surg_ = 0.019, *F*_surg(1,8)_ = 8.523; *P*_drug_ = 0.256, *F*_drug(1,8)_ = 1.499; *P*_int_ = 0.019, *F*_int(1,8)_ = 8.523), synapsin 1 (*P*_surg_ = 0.013, *F*_surg(1,8)_ = 10.257; *P*_drug_ = 0.030, *F*_drug(1,8)_ = 6.956; *P*_int_ = 0.021, *F*_int(1,8)_ = 8.254), PSD95 (*P*_surg_ = 0.003, *F*_surg(1,8)_ = 16.908; *P*_drug_ = 0.004, *F*_drug(1,8)_ = 15.535; *P*_int_ = 0.033, *F*_int(1,8)_ = 6.646), CaMKIIβ (*P*_surg_ = 0.037, *F*_surg(1,8)_ = 6.203; *P*_drug_ = 0.020, *F*_drug(1,8)_ = 8.463; *P*_int_ = 0.029, *F*_int(1,8)_ = 7.001), NR2A (*P*_surg_ < 0.001, *F*_surg(1,8)_ = 27.121; *P*_drug_ = 0.021, *F*_drug(1,8)_ = 8.224; *P*_int_ = 0.037, *F*_int(1,8)_ = 6.253), NR2B (*P*_surg_ < 0.001,*F*_surg(1,8)_ = 43.819; *P*_drug_ = 0.002, *F*_drug(1,8)_ = 21.952; *P*_int_ = 0.002, *F*_int(1,8)_ = 20.371), CaMKIIα (*P*_surg_ < 0.001, *F*_surg(1,8)_ = 64.159; *P*_drug_ < 0.001, *F*_drug(1,8)_ = 31.208; *P*_int_ < 0.001, *F*_int(1,8)_ = 47.622), and CaMKIIβ (*P*_surg_ < 0.001, *F*_surg(1,8)_ = 65.655; *P*_drug_ < 0.001, *F*_drug(1,8)_ = 25.505; *P*_int_ < 0.001, *F*_int(1,8)_ = 39.780).

## Discussion

In the present study, we found that the laparotomy-induced hippocampus-dependent LTM impairments were accompanied by the decreased acetylation levels of hippocampal histone H3 and H4 in 16- but not 3-month old mice. Treatment with SAHA rescued the histone acetylation levels and ameliorated the hippocampus-dependent LTM impairments in 16-month old mice exposed to the laparotomy.

Studies have revealed many risk factors, including advanced age, poor education, duration of anesthesia, respiratory complications, severity of coexisting illness, and psychoactive drugs, contributing to the development of POCD (Moller et al., 1998; Wan et al., 2007). Of these, advanced age is regarded as the prominent risk factor for the occurrence and development of POCD (Moller et al., 1998; Wan et al., 2007; Fidalgo et al., 2011). Therefore, aging animals were used to establish the relevant POCD models (Rosczyk et al., 2008; Li et al., 2014). We showed that 3-month old mice exposed to the laparotomy had no cognitive deficits, but 16-month old mice had the hippocampal LTM impairments, suggesting that the laparotomy can induce age-related behavioral impairments. Moreover, the context-dependent associative memory serves as an indicative of hippocampus-dependent associative memory and the tone-dependent associative memory requires proper function of amygdala (Barrientos et al., 2012). Thus, the impaired hippocampal LTM in 16- but not 3-month old mice indicated that the deteriorating effects of the laparotomy on the cognitive function are age-related. Similar to previous studies (Rosczyk et al., 2008; Li et al., 2014), we also did not observe an impaired STM in the 3- or 16-month old mice, which indicated that the model of POCD used in this study unaffected the intact STM.

Epigenetics have been shown to be deeply involved in the learning and memory deficits in neurodegenerative diseases (Govindarajan et al., 2011; Haettig et al., 2011; Haberman et al., 2012), whose phenotypes and pathogenesis are similar to POCD (Wang et al., 2013; Luo et al., 2014; Xu et al., 2014). However, the role of epigenetics in the development of POCD remains to be investigated. In our study, the decreased levels of hippocampal histone H3 and H4 acetylation and the impaired cognition in the 16-month old mice exposed to the laparotomy were prevented by the SAHA treatment, which suggested that the down-regulation of hippocampal acetyl-H3 and acetyl-H4 contributes to the pathogenesis of POCD. Moreover, SAHA did not up-regulate the histone acetyl-H3 and acetyl-H4 in the mice exposed to the sham surgery compared with those exposed to the laparotomy, which indicated that the over activity of HDAC makes it more sensitive to SAHA. Previous studies have revealed that not all lysines in histone proteins are affected in the development of neurodegenerative disorders (Miao et al., 2014; Zhong et al., 2014) and different diseases induce histone acetylation level changes at different sites (Guan et al., 2009; Itzhak et al., 2013). Our results showed that the laparotomy induced histone acetylation level changes at the sites of H3K9, H4K5, and H4K12, but not H3K14, which suggested that investigating these specific acetylation sites would be helpful to further understand the potential pathogenesis of POCD.

To clarify how the altered histone acetylation leads to the laparotomy-induced cognitive deficits, we investigated the expression of proteins related to cognition performance in 16-month old mice. Studies have linked the increased histone acetylation in the hippocampus to memory permissive for the transcription of learning-related plasticity genes (Ravi and Kannan, 2013) and attributed neuron damages and synaptic plasticity changes to the development of POCD (Bozon et al., 2002; Jungwirth et al., 2009; Cibelli et al., 2010; Lin and Zuo, 2011). We observed that neuroapoptosis-related proteins cleaved caspase-3 and iNOS were up-regulated, and synaptic plasticity-related proteins BDNF, synapsin 1, and PSD95 were down-regulated in the 16-month old mice after the laparotomy.

NMDAR and CaMKII proteins mainly locate at hippocampus and prefrontal cortex and play critical roles in learning and memory (Coultrap et al., 2014). Both the aged rats after the isoflurane/nitrous oxide anesthesia and the AD-like rats exhibit cognitive deficits and neuroapoptosis associated with an over-expression of hippocampal NR2B (Liu et al., 2012; Mawhinney et al., 2012). In aged mice, a higher expression of NMDAR2 was associated with poorer memory (Zhao et al., 2009). Therefore, our finding that the activation of NMDAR2-CaMKII pathway was observed in the hippocampus of 16-month old mice exposed to the laparotomy might correspond to their memory deficits. It was well studied that extra-synaptic NMDARs interfered with the BDNF expression, shut off cell survival pathway, induced mitochondrial dysfunction and activated pro-death molecules (Hardingham et al., 2002). Our results of reduced BDNF and increased cleaved caspase-3 and iNOS suggested that cell death might be triggered in our aging mice with POCD. Since SAHA alleviated the up-regulation of NR2, CaMKII, cleaved caspase-3 and iNOS, it was conceivable that the pathological high expression of these molecules contributed to the development of POCD possibly by histone acetylation.

Increased histone acetylation is generally associated with a chromatin structure that is more permissive for gene transcript. Our finding of the up-regulations of iNOS, NR2A, NR2B and CaMKII in the laparotomy with lower acetylation indicated that the expressions of these genes might not be regulated directly through histone acetylation on their promoters. It might have other regulation mechanisms following histone acetylation underlying these genes. While the down-regulation of BDNF, synapsin 1 and PSD 95 corresponding to the down-regulation of histone acetylation indicated that the promoters of these genes might be deacetylated by HDAC in our POCD model. Further studies on the promoters of these genes by chromatin immunoprecipitation are needed to investigate the dynamic state of histone acetylation associated with these changes of gene expressions.

It is clear that not only cardiac surgeries, but also abdominal orthopedic can also produce POCD (Martin et al., 2005). The possible reason is that immune challenge induced by the laparotomy results in an exaggerated inflammatory response in the hippocampus, a region of the brain that contains a large number of pro-inflammatory cytokine receptors, through the communication between the peripheral immune system and the brain (Parnet et al., 1994). The neuroinflammatory response in turn affect the expression of other important genes to cause cognitive dysfunction, which is associated with epigenetic changes such as histone acetylation or deacetylation. At an advanced age, the long lasting neuroinflammatory response likely plays an important role to cause hippocampal-dependent memory deficits (Maier, 2003; Barrientos et al., 2009). That explains the facts that age is the strongest risk factor for the development of POCD, and that anti-inflammatory is considered as a viable strategy to prevent POCD. Noticeably, if inflammation does not subside, it can contribute to the pathogenesis of disease (Vacas et al., 2013). Our findings of decreased histone acetylation in POCD provide novel insight into the pathology of POCD and important preclinical evidences supporting that SAHA may serve as a potential therapeutic agent for POCD.

## Conflict of Interest Statement

The authors declare that the research was conducted in the absence of any commercial or financial relationships that could be construed as a potential conflict of interest.

## Abbreviations

POCD: postoperative cognitive dysfunction
HATs: histone acetyltransferases
HDACs: histone deacetylases
SAHA: suberoylanilide hydroxamic acid
DMSO: dimethylsulfoxide
iNOS: inducible nitric oxide synthase
BDNF: brain-derived neurotrophic factor
PSD95: postsynaptic density 95
NMDA: N-methyl-D-aspartate
CaMKII: calcium/calmodulin dependent kinase II
HE staining: Hematoxylin-eosin staining
PBS: phosphate-buffered saline
PFA: phosphate-buffered paraformaldehyde
STM: short-term memory
LTM: long-term memory
TBST: Tris-Buffered Saline Tween
Ac-H3K9: histone H3(acetyl K9)
Ac-H3K14: histone H3(acetyl K14)
Ac-H4K5: histone H4 (acetyl K5)
Ac-H4K12: histone H4 (acetyl K12)
Ac-H3: acetyl histone H3
Ac-H4: acetyl histone H4 (Lys5/8/12/16)
GAPDH: genes glyceraldehyde-3-phosphate dehydrogenase
real-time PCR: real-time polymerase chain reaction
AD: Alzheimer’s disease
LTD: long-term depression
LTP: long-term potentiation.
